# Metabolic cooperation between conspecific genotypic groups contributes to bacterial fitness

**DOI:** 10.1038/s43705-023-00250-8

**Published:** 2023-04-28

**Authors:** Lin Lin, Rubing Du, Qun Wu, Yan Xu

**Affiliations:** Key Laboratory of Industrial Biotechnology of Ministry of Education, State Key Laboratory of Food Science and Technology, Laboratory of Brewing Microbiology and Applied Enzymology, School of Biotechnology, Jiangnan University, Wuxi, Jiangsu 214122 China

**Keywords:** Microbiology, Microbial genetics

## Abstract

Microbial interactions are important for the survival of species and the stability of the microbial ecosystem. Although bacteria have diverse conspecific genotypes in the natural microbial ecosystem, little is known about whether wild-type strains within species would interact with each other and how the intraspecific interaction influences the growth of the species. In this work, using *Lactobacillus acetotolerans*, a dominant species with diverse conspecific genotypes in natural food fermentation ecosystems as a case, we studied the interactions between different genotypic groups of this species. In interspecific and intraspecific pairwise cocultures, the growth of *L. acetotolerans* decreased, but the increase of the phylogenetic similarity would reduce this negative effect, indicating a potential intraspecific interaction of this species. Meanwhile, the strain classification method affected the analysis of intraspecific interactions, which can be efficiently demonstrated using 99.5% average nucleotide identity (ANI) as the strain-level classification method. Using this ANI classification method, we revealed the population fitness significantly increased in cocultures of different genotypic groups. Facilitation involving 11 amino acids was identified between different ANI genotypic groups, which was beneficial for increasing population fitness. This work revealed that wild-type conspecific strains could interact with each other via cooperative metabolic changes and benefit each other to increase fitness. It shed new light on the survival and stability of species in natural microbial ecosystems.

## Introduction

Microbial communities are complex multispecies assemblages characterised by a multitude of microbial interactions [1, 2]. Interactions between species can be positive and promote the growth of another species by increasing the availability of nutrients or creating new niches [3]. There are also negative interactions between species that inhibit the growth of other species by competing for nutrients or by producing antibiotics, including bacteriocins, toxins, and peptides [4]. These microbial interactions form and stabilise microbial ecosystems and contribute to the adaptation of members to the environment [5, 6]. As a result, revealing the complex microbial interactions would be important for understanding the survival and stability mechanisms of microbes in the microbial ecosystem.

Interactions have been widely reported to occur between different microbial species [7]. However, it is controversial whether species act as the smallest unit in microbial ecosystems, because different individuals within the same species may have different functions in the environment [8]. At present, studies have revealed a variety of intraspecific differences in microbial genomes and phenotypes [9, 10]. As a result, different strains within the same species are exposed to each other for a long time in the same environment and may interact with each other more frequently [11]. Intraspecific variation may influence community composition and function as much as variation among species [12]. These interactions also play important roles in the stability of microbial communities [13, 14], although they are less studied than interspecific interactions.

Lactic acid bacteria play a prominent role in the worldwide food supply, including in the production of cheese, wine, sourdough, Chinese liquor and various other indigenous food fermentations [15–17]. Lactobacilli are an important component of lactic acid bacteria. In this work, we hypothesised that intraspecific interactions occur between different wild-type strains, and the average nucleotide identity (ANI) level classification would affect the intraspecific interaction results. To find the most useful ANI level that distinguishes ecologically distinct groups of the species for intraspecific interaction analysis, we chose one representative lactobacilli species based on the amplicon sequencing data from 558 food fermentation samples. Using a widely distributed lactic acid bacterial species with high intraspecific diversity, we compared the effects of ANI-level classification on intraspecific interactions, and analysed the type of interaction between different conspecific strains and elucidated a metabolic cooperation (a novel kind of ecological facilitation where one genotypic group can alter the metabolism of the other, and benefit from this metabolic variation) scheme among different conspecific genotypic groups.

## Materials and methods

### Amplicon sequencing data collection and processing

The amplicon sequencing data of V3-V4 hypervariable region of the 16S rRNA gene from food fermentations were downloaded from the National Center for Biotechnology Information (NCBI) SRA database and DNA Data Bank of Japan (DDBJ) using fastq-dump (version 2.10.8), which included 558 samples (Supplementary Table 1). All sequencing data were produced with Illumina platforms for paired-end sequencing, and different primer pairs were used (Supplementary Table 1). The raw data was processed using VSEARCH [18]. Briefly, the forward and reversed sequences were merged with default parameters. Merged sequences with an error rate > 0.01 were removed. Then, the clean merged sequences were processed using QIIME2 [19]. The amplicon sequence variant (ASV) and ASV table were generated by DADA2 pipeline with default parameters [20]. The taxonomic classification of each ASV was annotated via aligning ASV’s sequence to NCBI NR database using BLAST program.

### Collection of different bacterial strains

*Lactobacillus acetotolerans* was isolated using cereal fermentation samples (Chinese liquor fermentations, vinegar fermentations, and sour dough products). This species is difficult to cultivate due to its extremely slow growth. We used modified Man Rogosa Sharpe (MRS) medium for the isolation. MRS medium was supplemented with 1% fructose, 0.5% corn starch, and 0.001% DL-mevalonolactone, and the pH was adjusted to 3.85 to obtain modified MRS medium. For the isolation of *L. acetotolerans*, 5 g fermented samples were mixed with 100 mL of sterile saline solution, and serial dilutions were prepared. The diluted solutions were spread onto modified MRS agar plates and cultured under anaerobic conditions at 30 °C. Extremely small colonies were finally observed on the medium after 10 days of culture. A total of 210 clones were selected, and only 17 isolates were identified as *L. acetotolerans*.

Eleven other bacterial strains were isolated from Chinese liquor fermentations. *Lactobacillus delbrueckii*, *Companilactobacillus crustorum*, *Lactobacillus plantarum*, *Lentilactobacillus hilgardii*, *Lacticaseibacillus paracasei*, *Limosilactobacillus fermentum*, and *Levilactobacillus brevis* were deposited in Laboratory of Brewing Microbiology and Applied Enzymology, Jiangnan University, with accession numbers of LBM 10007 [21], LBM 10004 [21], LBM 10005 [21], LBM 12050, LBM 12051, LBM 12052, LBM 10003 [21], respectively. *Lentilactobacillus buchneri*, *Pediococcus acidilactici*, *Weissella paramesenteroides*, and *Bacillus amyloliquefaciens* were deposited in China General Microbiological Culture Collection Center with accession numbers of CGMCC 14271 [22], CGMCC 22237, CGMCC 22238, CGMCC 12593 [23], respectively.

### DNA extraction and whole-genome sequencing

The genomic DNA of *L. acetotolerans* and other bacterial strains was extracted using a Rapid Bacterial Genomic DNA Isolation Kit (Sangon Biotech, Shanghai, China). Then, a sequencing library was prepared with the high-molecular-weight DNA using a Rapid Sequencing Kit (SQK-RAD003) (Oxford Nanopore Technologies, Ltd., Oxford, UK).

### Gene prediction and phylogenetic analysis of whole genomes

The sequencing reads of genomes were selected based on size and quality using Filtlong v0.2.0. The obtained reads were assembled *de novo* using Canu [24]. Genomes were circularised using Circlator-1.4.1. Gene prediction and computational annotation of protein-coding genes (CDSs) were performed using Prodigal [25]. Functional annotation was performed using the Kyoto Encyclopedia of Genes and Genomes (KEGG) annotations [26]. We applied Cluster of Orthologous Groups of proteins (COG) databases [27] (BLASTP cut-off, E value < 10^−5^) for genome annotation. Pangenome analysis was based on the Bacterial Pan Genome Analysis (BPGA) tool [28]. FastANI [29] was used to calculate ANI value. We used whole–genome sequences to construct the amino acid biosynthesis pathway of *L. acetotolerans*, according to the KEGG and MetaCyc Metabolic Pathway Database.

### Monoculture and coculture experiments

We conducted 3 sets of mono- and coculture experiments. In the first culture series, 9 isolates were used for mono- and cocultures. For monocultures of 9 isolates, the initial concentration of each isolate was 1 × 10^6^ CFU/mL. For cocultures, different combinations of 2–9 isolates were mixed at the same concentrations to a total cell density of 1 × 10^6^ CFU/mL. We obtained a total of 95 culture trials (Supplementary Table 2), including 9 monoculture and 86 coculture trails, which were randomly generated by Xlstat in Excel [30]. Monoculture and coculture experiments were performed in 1.5 mL of chemically defined medium in 96-well plates under static and anaerobic conditions at 30 °C for 8 days, and the final bacterial solution was collected for biomass determination. The chemically defined medium contained a total of 57 components, and the 20 proteinogenic amino acids acted as the only organic nitrogen sources among 57 components [31]. Each culture had four biological replicates.

In the second culture series, 4 ASVs of *L. acetotolerans* and 11 other bacterial strains were used for mono- and pairwise coculture in 2 mL of chemically defined medium in a Transwell plate (12-well polystyrene plates with 0.4-μm pore size, Corning, NYC, NY, USA) (Supplementary Fig. 1) to separate different cells and allow the medium to pass through membrane pores. For the monoculture, *L. acetotolerans* was inoculated in both the upper and lower chambers of the Transwell plate, and the initial total concentration was 1 × 10^6^ CFU/mL. For the pairwise coculture, *L. acetotolerans* was cultured in the lower chamber, and 11 other strains were separately cultured in the upper chamber, and the initial concentrations of *L. acetotolerans* and other strain were each 1 × 10^6^ CFU/mL. All cultures were under static and anaerobic conditions at 30 °C for 8 days, and the final bacterial solution in the lower chamber was collected to determine the biomass of *L. acetotolerans*. Each culture had four biological replicates.

In the third culture series, three genotypic groups were used for mono- and cocultures. We performed two groups of culture experiments, one group contained isolate_1 (ANI_I), isolate_4 (ANI_II) and isolate_9 (ANI_III), and the other contained isolate_3 (ANI_I), isolates_8 (ANI_II), and isolates_9 (ANI_III). Each experimental group contained monoculture, pairwise and three-genotype cocultures, and cultures were performed in 2 mL of chemically defined medium in Transwell plates. For the monoculture, three genotypic groups were separately inoculated in both the upper and lower chambers of the Transwell plate, and the total initial concentration was 1 × 10^6^ CFU/mL. For the pairwise coculture, two genotype groups were separately cultured in the upper and lower chambers. For the three-genotype coculture, one genotype was separately inoculated in the lower chamber, and the other two groups were inoculated in the upper chamber. For all cocultures, different isolates were inoculated at the same concentrations to a final total cell density of 1 × 10^6^ CFU/mL. The cultures were grown under static and anaerobic conditions at 30 °C for 8 days. We collected only cells in the lower chamber to determine the biomass and transcription levels. The bacterial solution was collected on Days 0, 2, 4, 6 and 8 for biomass and amino acid determination, and collected on Day 4 for transcriptional analysis. Each culture had four biological replicates.

### Culture experiments with conditioned medium

The conditioned medium was the mixture of the spent chemically defined medium and fresh chemically defined medium (1:1, v/v). Isolate_1, isolate_4 and isolate_9 represent the ANI_I, ANI_II and ANI_III groups, respectively, and isolate_3, isolate_8, and isolate_9 represent the ANI_I, ANI_II and ANI_III groups, respectively. Each isolate from a single genotypic group of *L. acetotolerans* was inoculated into both the upper and lower chambers and cultured in 2 mL of chemically defined medium in a Transwell plate under static and anaerobic conditions at 30 °C for 4 days. The bacterial solution was collected and centrifuged at 11,180 × *g* for 10 min at 4 °C. The supernatant was filtered through a 0.22-µm membrane filter, and the filtrate was used as the spent chemically defined medium.

*L. acetotolerans* was inoculated into conditioned medium at an initial concentration of 1 × 10^6^ CFU/mL. After statically and anaerobically culturing at 30 °C, the bacterial solution was collected on Days 0, 2, 4, 6 and 8 to determine the amino acid content. Each culture had four biological replicates.

### Microbial quantification

The biomass of *L. acetotolerans* was determined by measuring the optical density at 600 nm (OD_600_) using a BioTek Synergy microplate reader (BioTek, Winooski, VT, USA) and real-time quantitative PCR (qPCR) performed on a Real-Time PCR System (Applied Biosystems, Foster City, CA, USA). For qPCR, the helix-turn-helix transcriptional regulator gene (GenBank BAQ57713.1) of *L. acetotolerans* were amplified using the primers LaF (5’ - AAAAAGCAGAGTGGAGAAAATACT - 3’) and LaR (5’ - CCAATAAAAAGAGCAACAGCA - 3’) [21]. The standard curve of the primers was y = −3.804x + 43.816 (R^2^ = 0.9996). qPCR was performed as previously described [22].

### RNA extraction, sequencing and analysis

Total RNA was extracted using an RNA extraction kit (Tiangen, Beijing, China). rRNA was depleted with the Ribo-zero TM rRNA Removal Kit (Epicenter Biotechnologies, Madison, WI, USA), and sequencing libraries were prepared using the NEBNext^®^ Ultra II Directional RNA Library Prep Kit (New England Biolabs, Ipswich, MA, USA). Sequenced reads were aligned to whole genomes using Bowtie2 [32], and HTSEq [33] was used for quantitative analysis of each CDS in the genome. Gene expression levels were estimated by fragments per kilobase of transcript per million fragments mapped (FPKM) by RNA-Seq by Expectation-Maximization (RSEM) software [34]. Genes with false discovery rate-adjusted *p* values of <0.05 and an absolute fold change of >2 were identified as significantly differentially expressed genes [35]. The gene transcription results were used to construct the biosynthesis pathways of amino acids according to the KEGG pathway database and MetaCyc Metabolic Pathway Database.

### Amino acid quantification

The collected bacterial solution was passed through a 0.22-µm membrane filter and diluted with 10% trichloroacetic acid at a ratio of 1:1, and then 0.5 mL was used for amino acid analysis on a high-performance liquid chromatograph (Agilent Technologies, Santa Clara, CA, USA) according to a previously reported protocol [36].

### Fitness calculation

The relative fitness of selected isolates was measured in mono- and cocultures. Relative fitness was measured from the final and initial biomass of isolate based on qPCR analysis (Eq. (1)). The relative fitness of the monoculture was set as 1 [37].

1 $${{{{{{{\mathrm{Relative}}}}}}}}\,{{{{{{{\mathrm{fitness}}}}}}}} = \frac{{{{{{{{{\mathrm{Log}}}}}}}}_2\frac{{{{{{{{{\mathrm{Q}}}}}}}}_{{{{{{{{\mathrm{final}}}}}}}} - {{{{{{{\mathrm{co}}}}}}}}}}}{{{{{{{{{\mathrm{Q}}}}}}}}_{{{{{{{{\mathrm{initial}}}}}}}}}}}}}{{{{{{{{{\mathrm{Log}}}}}}}}_2\frac{{{{{{{{{\mathrm{Q}}}}}}}}_{{{{{{{{\mathrm{final}}}}}}}} - {{{{{{{\mathrm{mon}}}}}}}}}}}{{{{{{{{{\mathrm{Q}}}}}}}}_{{{{{{{{\mathrm{initial}}}}}}}}}}}}}$$

Q_final-co_ represents the average biomass of the isolate in cocultures, Q_final-mono_ represents the average biomass of the isolate in monocultures, and Q_initial_ represents the initial biomass of the isolate.

The relative amino acid production was calculated using Eq. (2).

2 $${{{{{{{\mathrm{Relative}}}}}}}}\,{{{{{{{\mathrm{amino}}}}}}}}\,{{{{{{{\mathrm{acid}}}}}}}}\,{{{{{{{\mathrm{production}}}}}}}} = \frac{{{{{{{{{\mathrm{C}}}}}}}}_{{{{{{{{\mathrm{final}}}}}}}} - {{{{{{{\mathrm{con}}}}}}}}}}}{{{{{{{{{\mathrm{C}}}}}}}}_{{{{{{{{\mathrm{initial}}}}}}}} - {{{{{{{\mathrm{con}}}}}}}}}}}$$

C_final-con_ represents the final average concentration of amino acid in cultures with conditioned medium (Day 8), and C_initial-con_ represents the initial average concentration of amino acid in cultures with conditioned medium (Day 0).

### Statistics

Independent sample *t-*tests were used to assess the differences in biomass, amino acid concentrations between monocultures and cocultures, and differences of amino acid concentrations. The Pearson correlation coefficient (r) between relative fitness and ANI level was calculated by linear regression analysis using Origin (OriginLab, Northampton, MA, USA). A value of *p* < 0.05 was considered statistically significant. The averages of four biological replicates are reported ±their standard deviations.

## Results

### Phylogenetic similarity influenced relative fitness of *Lactobacillus acetotolerans* in the interspecific and intraspecific cocultures

To assist in choosing one representative lactobacilli species for revealing the potential microbial interactions between lactobacilli species, the bacterial composition from 558 food fermentation samples were analysed (Supplementary Table 1). Lactobacillaceae was the most frequent family (distribution frequency, 91%) in the bacterial community across all food fermentation samples (Supplementary Fig. 2A). The highest number of Lactobacillaceae species in fermentations reached 28 (Supplementary Fig. 2B). *L. acetotolerans* dominated at the species level (relative abundance >1% in Lactobacillaceae) in 38% of food fermentations, and the highest relative abundance reached 98% in Lactobacillaceae (Supplementary Fig. 2C). Moreover, the intraspecific diversity of *L. acetotolerans* (10 ASV types) was highest among Lactobacillaceae in food fermentations (Supplementary Fig. 2D). As a result, *L. acetotolerans* was selected as a target species to analyse the interspecific and intraspecific interactions in food fermentations.

**Fig. 1:**
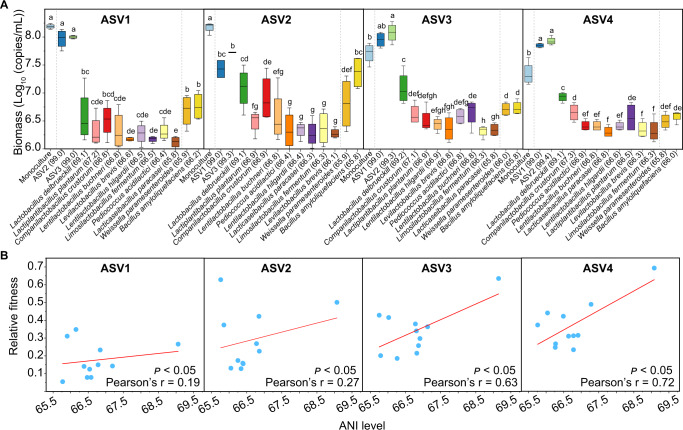
Relative fitness and biomass of different *L. acetotolerans* isolates in pairwise cocultures. **A** Biomass of different *L. acetotolerans* isolates from 4 ASV types in pairwise cocultures with 11 other bacterial species. The other species cocultured with *L. acetotolerans* are indicated on the abscissa, and the number in the brackets after the species name indicates the ANI level (%) between *L. acetotolerans* isolate and other species. All cultures were under static and anaerobic conditions at 30 °C, and the biomass were determined on Day 8. Each culture had four biological replicates. Unlike letters indicate significant differences (*t*-test, *p* < 0.05). **B** Correlation of relative fitness of *L. acetotolerans* and ANI level between *L. acetotolerans* and 11 other species in pairwise cocultures. The red line represents fitted curve between relative fitness and ANI level.

We obtained 17 *L. acetotolerans* isolates of which 9 belonged to ASV1, 1 belonged to ASV2, 2 belonged to ASV3 and 5 belonged to ASV4. We also obtained 11 different strains belonging to 11 other species in food fermentations, and their ANI levels with *L. acetotolerans* ranged from 65.8% to 69.2% (Fig. 1A). We analysed intraspecific interactions between 4 *L. acetotolerans* isolates (each from one ASV) and interspecific interactions of *L. acetotolerans* isolates with 11 other strains. The growth of *L. acetotolerans* decreased in pairwise cocultures with other species, but this growth reduction weakened with the increase of phylogenetic similarity between the coculture partners in Lactobacillaceae family, except for *W. paramesenteroides* (Fig. 1A). The relative fitness was significantly positively correlated with ANI level between *L. acetotolerans* and 11 other species (Fig. 1B). In addition, the fitness of *L. acetotolerans* was highest in the intraspecific coculture among all cocultures. The relative fitness of ASV3 and ASV4 significantly increased in coculture with ASV1 or ASV2 strains. It suggested a potential intraspecific interaction between *L. acetotolerans* strains.

### Relatedness of *Lactobacillus acetotolerans* strains affected the intraspecific interactions

To reveal the potential intraspecific interactions between *L. acetotolerans* strains, we classified *L. acetotolerans* isolates by different levels of relatedness, and analysed the effects of the strain classification criterion on intraspecific interactions. We analysed the genomic characteristics of different *L. acetotolerans* isolates. We chose 3 isolates each from ASV1 and ASV4, and all isolates from ASV2 (1 isolate) and ASV3 (2 isolates) for genome sequencing (Table 1). The genome sizes of these 9 isolates ranged from 1.49 Mbp to 1.62 Mbp. The number of genes in these 9 isolates presented significant differences, ranging from 1426 to 2291 (Table 1, Supplementary Fig. 3).

**Table 1:** Genome features of 9 *L. acetotolerans* isolates.

Isolate	ASV	ANI^a^	Genome size (Mbp)	GC%	Gene numbers
Isolate_1	ASV1_1	ANI_I	1.62	36.8	1878
Isolate_2	ASV1_2	ANI_I	1.61	36.8	1878
Isolate_3	ASV1_3	ANI_I	1.61	36.8	2291
Isolate_4	ASV3_1	ANI_II	1.53	36.6	1695
Isolate_5	ASV3_2	ANI_II	1.55	36.3	1674
Isolate_6	ASV4_1	ANI_II	1.54	36.5	1426
Isolate_7	ASV4_2	ANI_II	1.53	36.6	1683
Isolate_8	ASV4_3	ANI_II	1.53	36.6	1704
Isolate_9	ASV2_1	ANI_III	1.49	36.7	1624

^a^ANI group was classified by 99.5% average nucleotide identity based on whole–genome sequences.

**Fig. 2:**
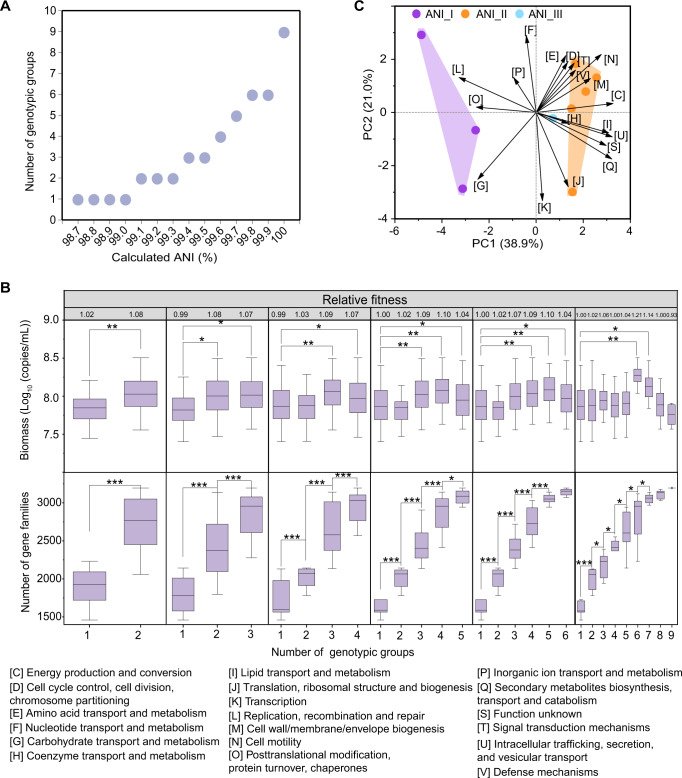
Effect of strain classification method on intraspecific interactions of *L. acetotolerans*. **A** Classification of genotypic groups of *L. acetotolerans* under different ANI levels. The lowest ANI level among 9 isolates was 98.7%. **B** Relative fitness, biomass (qPCR) and number of gene families in mono- and cocultures of different genotypic groups in 6 classification methods according to different ANI levels. Difference of biomass in 95 trials containing 9 monocultures and 86 cocultures (Supplementary Table 2). Cultures were performed in 1.5 mL of chemically defined medium in 96-well plates under static and anaerobic conditions at 30 °C for 8 days. Error bars indicate the standard deviation from four biological replicates. Asterisks indicate significant differences of biomass and number of gene families in coculture and monoculture (*t*-test, *p < 0.05, **p < 0.01, ***p < 0.001). **C** The variation in the gene contents of all COG categories in three ANI groups (classified by 99.5% average nucleotide identity) compared by principal component analysis.

To analyse the effect of strain classification method on intraspecific interactions, we used these 9 isolates for monoculture and coculture by randomly combining them, and a total of 95 trials were performed (Supplementary Table 2). ANI classification has appeared to offer finer resolution between conspecific strains [29]. To analyse the effect of strain classification on intraspecific interactions, we calculated the pairwise ANI for each genome (Supplementary Fig. 4), and classified 9 isolates into different ANI groups according to different ANI cut-off values. We obtained 6 different classification methods (Fig. 2A). Regardless of which classification method was used, the fitness was improved after coculture, except for the 9-group classification method (100% ANI level). Only the 2- and 3-group classification methods (99.1–99.5% ANI level) revealed significant intraspecific interactions, in which the combinations of different ANI groups all presented significant differences from those of single ANI groups (*p* < 0.05) (Fig. 2B). It indicated that the intraspecific classification method at different ANI level affected the intraspecific interaction result, and that 99.5% might be a good cut-off level for strain classification when studying microbial intraspecific interactions.

By genome-wide analysis, the number of gene families were calculated in the combination of ANI groups under different classification methods (Fig. 2B). The average number of gene families increased when different ANI groups were combined under the 2-, 3-, 4- and 5- group classification methods, indicating that coculturing different ANI groups might cause functional complementarity among different ANI groups. Taking 99.5% ANI as the cut-off value, 9 isolates were classified into three genotypic groups (Table 1). ASV1 was classified into ANI_I, ASV3 and ASV4 were classified into ANI_II, and ASV2 was classified into ANI_III. The principal component analysis of the gene contents in each COG category indicated that all three ANI groups were different from each other (Fig. 2C). The gene compositions of the top two categories (carbohydrate transport and metabolism; amino acid transport and metabolism) were also different among the three genotypic groups (Supplementary Fig. 5A, B). This result indicated that classification based on 99.5% ANI level would be suitable for strain classification. In addition, ANI_I-type isolates grew quickly and had the highest final average biomass (8.10 ± 0.20 (log_10_ (copies/mL)), followed by the ANI_III-type isolate (8.00 ± 0.15 (log_10_ (copies/mL)), and ANI_II-type isolates grew the slowest and had the lowest final average biomass (7.77 ± 0.31 (log_10_ (copies/mL)) (Supplementary Fig. 6). It indicated the growth difference among these different genotypic groups.

### Intraspecific interactions between genotypic groups increase fitness

To deeply analyse the intraspecific interactions, we chose isolates in each ANI group for coculture. We performed two groups of coculture experiments, one containing isolate_1 (ANI_I), isolate_4 (ANI_II), and isolate_9 (ANI_III) and the other containing isolate_3 (ANI_I), isolate_8 (ANI_II), and isolate_9 (ANI_III), in Transwell plates to separate different isolates (Supplementary Fig. 1).

**Fig. 3:**
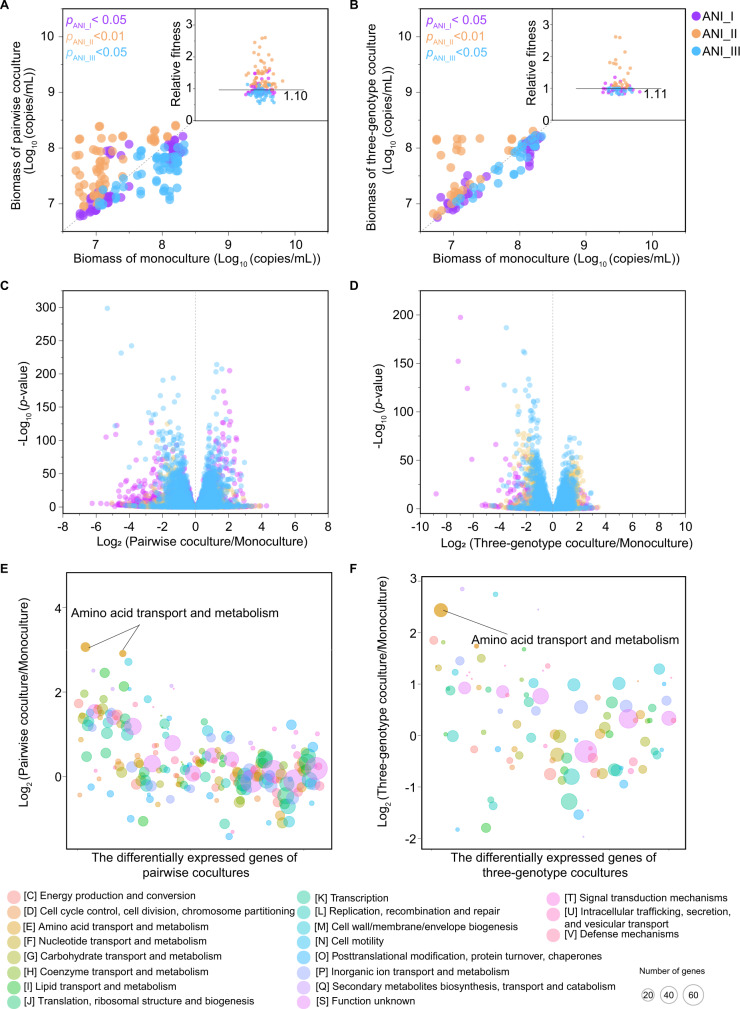
Intraspecific interactions between different genotypic groups. The biomass of different ANI groups in pairwise cocultures compared to that in monocultures (**A**) and in three-genotype cocultures compared to that in monocultures (**B**). Each dot indicates the biomass of one isolate in the monoculture (abscissa) and in the coculture (ordinate) in the Transwell plate (Supplementary Fig. 1). In the Transwell plate, cells of different genotypes can be separated in upper and lower chambers, and the medium can pass through membrane pores between two chambers. Volcano plots showing gene expression data of three ANI groups in pairwise cocultures compared to that in monocultures (**C**) and in three-genotype cocultures compared to that in monocultures (**D**). Each dot indicates the transcription of one gene in one isolate. The log_2_ (gene transcription value in cocultures compared to that in monocultures) is shown on the abscissa, and the log_10_ (*p-*value) is shown on the ordinate. The colour of the dot represents the ANI group. The log_2_ fold change of differentially expressed genes (adjusted *p* < 0.05, |log_2_ fold change| > 1) in pairwise cocultures compared to that in monocultures (**E**) and in three-genotype cocultures compared to that in monocultures (**F**) under the COG category. Each bubble indicates one COG category in one ANI group. The average log_2_ (gene transcription value in the coculture compared to that in the monoculture) in the COG category are shown on the ordinate. The colour of the bubble represents the COG category, and the size of the bubble represents the number of differentially expressed genes in that COG category. Data were from two coculture experiment groups (isolate_1, isolate_4, and isolate_9; isolate_3, isolate_8, and isolate_9). Cultures were performed in Transwell plates under static and anaerobic conditions at 30 °C. The bacterial solution was collected on Days 2, 4, 6 and 8 for biomass and on Day 4 for transcription analysis. All data are based on four biological replicates.

We analysed the effect of intraspecific interactions on fitness using these different ANI groups. The biomass of ANI_I and ANI_III significantly decreased in pairwise and three-genotype cocultures (*t*-test, *p*_ANI_I_ < 0.05, *p*_ANI_III_ < 0.05), and the average fitness of ANI_I and ANI_III decreased to 0.82 and 0.56 in pairwise cocultures, and to 0.83 and 0.85 in three-genotype cocultures, respectively. The biomass of ANI_II significantly increased in these cocultures (*t*-test, *p*_ANI_II_ < 0.01), and the average fitness of ANI_II increased to 2.56 in pairwise cocultures and 2.03 in three-genotype cocultures (Fig. 3A, B).

We hypothesised that these intraspecific interactions might be due to induced changes in metabolism because there was no cell contact in the coculture with the Transwell plate. We analysed the gene transcription patterns of cocultures, as well as those of monocultures, of different ANI groups (Fig. 3C, D, Supplementary Fig. 7). In the two coculture experiment groups, the highest number of differentially expressed genes (adjusted *p* < 0.05, |log_2_ fold change| > 1) was 299 in ANI_I, 182 in ANI_II, 369 in ANI_III, in pairwise cocultures compared to monocultures (Fig. 3C, Supplementary Table 3). The highest number of differentially expressed genes (adjusted *p* < 0.05, |log_2_ fold change| > 1) was 133 in ANI_I, 358 in ANI_II, 267 in ANI_III, in the three-genotype cocultures compared to monocultures (Fig. 3D, Supplementary Table 3).

Differentially expressed genes in coculture were annotated according to COG categories (Fig. 3E, F). In pairwise cocultures, amino acid transport and metabolism was the most differentially expressed COG category in ANI_I (average log_2_ fold change = 3.07, number of differentially expressed genes = 18) and ANI_III (average log_2_ fold change = 2.72, number of differentially expressed genes = 10) (Fig. 3E). In the three-genotype coculture, secondary metabolite biosynthesis, transport and catabolism (average log_2_ fold change = 2.85, number of differentially expressed genes = 2) was the most differentially expressed COG category in ANI_II, and cell wall/membrane/envelope biogenesis (average log_2_ fold change = 2.75, number of differentially expressed genes = 3) and amino acid transport and metabolism (average log_2_ fold change = 2.44, number of differentially expressed genes = 28) were the most differentially expressed COG categories in ANI_I. The number of differentially expressed genes was the highest in the amino acid transport and metabolism category (Fig. 3F). These results indicated that amino acid transport and metabolism might be affected by intraspecific interactions in the coculture.

### Metabolic cooperation between conspecific genotypic groups led to an increase in bacterial fitness

We deduced that the improvement of fitness might be related to metabolic exchange of amino acids. A total of 18 kinds of amino acids were detected in the fermentations (Supplementary Fig. 8). Compared with those in monocultures, all 18 amino acids varied in both pairwise and three-genotype cocultures (Supplementary Fig. 8). This result indicated the potential metabolic exchange of amino acids between different genotypic groups within species.

To explore whether the isolates have the potential to synthesise amino acids, we used whole–genome sequences to construct the amino acid biosynthesis pathway of *L. acetotolerans* (Supplementary Fig. 9). The genomic results showed that the three genotypic groups all have the potential to synthesise all these amino acids.

In addition, in order to determine the effect of coculture on amino acid production, we determined the concentrations of amino acids of each genotypic group cultured with conditioned medium from the other genotypic group, and calculated the relative amino acid production level of this genotypic group. A relative amino acid production level of the genotypic group that was more than 1 (*t*-test, *p* < 0.05) indicated that this genotypic group might increase the production of this amino acid. A level that was less than 1 (*t*-test, *p* < 0.05) indicated that this genotypic group might utilise this amino acid.

**Fig. 4:**
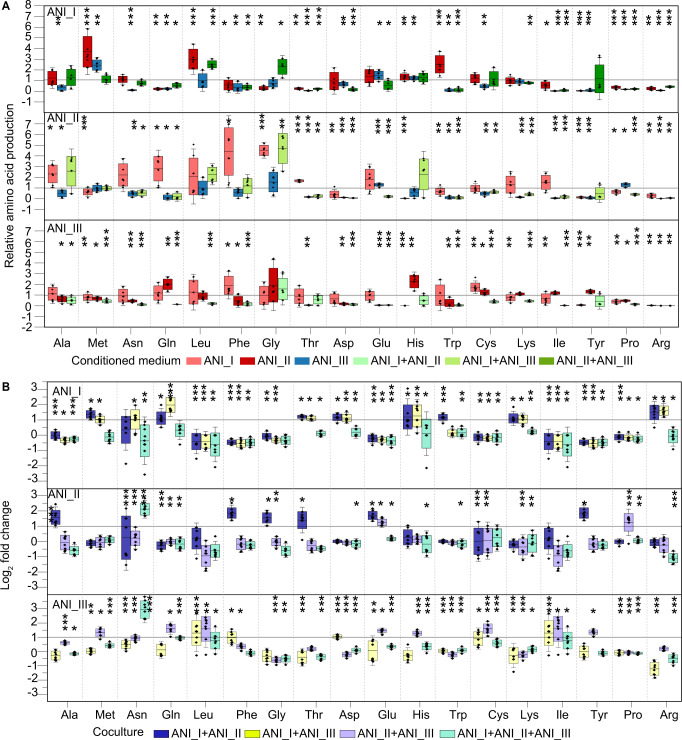
Relative amino acid production in cultures with conditioned medium and the transcription of amino acid-related genes in cocultures. **A** The relative amino acid production of the ANI _I, ANI_II, and ANI_III groups in conditioned medium from the other genotypic groups. Scatter points indicate the relative production of amino acids of one isolate in conditioned medium. **B** The transcription of the last gene in the biosynthesis pathway of different amino acids in ANI _I, ANI_II, and ANI_III groups in coculture with other genotypic groups. Scatter points indicate the log_2_ fold change level of transcriptional data of one isolate in coculture compared to monoculture. All the data were from two coculture experiment groups (isolate_1, isolate_4, and isolate_9; isolate_3, isolate_8, and isolate_9). Cultures were performed in Transwell plates (Supplementary Fig. 1) under static and anaerobic conditions at 30 °C. In the Transwell plate, cells of different genotypes can be separated in upper and lower chambers, and the medium can pass through membrane pores between the two chambers. The bacterial solution was collected on Days 0 and 8 for amino acid analysis in cultures with conditioned medium and on Day 4 for transcription analysis in cocultures. Error bars indicate the standard deviation of four biological replicates; asterisks represent significant differences in relative amino acid production between Day 8 and Day 0 (*t*-test, *p < 0.05, **p < 0.01, ***p < 0.001), and significant differences in gene transcription between coculture and monoculture (* adjusted *p* < 0.05, ** adjusted *p* < 0.01, *** adjusted *p* < 0.001, |log_2_ fold change| > 1).

When ANI_I was cultured in ANI_II conditioned medium, the relative amino acid production levels of methionine, leucine, histidine and tryptophan were more than 1 (*t*-test, *p* < 0.05) in ANI_I (Fig. 4A and Supplementary Fig. 10A). The transcriptions of genes related to methionine, glutamine, threonine, aspartate, histidine, tryptophan, lysine, and arginine were upregulated (adjusted *p* < 0.05, log_2_ fold change > 1) in ANI_I in the coculture with ANI_II (Fig. 4B). Moreover, when ANI_II was cultured in ANI_I conditioned medium, the relative amino acid production levels of methionine, aspartate, histidine, tryptophan, tyrosine, proline, and arginine were less than 1 (*t*-test, *p* < 0.05) in ANI_II (Fig. 4A and Supplementary Fig. 10B). This result confirmed a metabolic cooperation related to methionine, histidine and tryptophan, where the ANI_I group increased the production of these amino acids and the ANI_II group utilised these amino acids (Supplementary Table 4).

When ANI_II was cultured in ANI_I conditioned medium, the relative amino acid production levels of alanine, glutamine, phenylalanine, glycine, and threonine were more than 1 (*t*-test, *p* < 0.05) in ANI_II (Fig. 4A and Supplementary Fig. 10B). The transcriptions of genes related to alanine, phenylalanine, glycine, threonine, glutamate, and tyrosine were upregulated (adjusted *p* < 0.05, log_2_ fold change > 1) in ANI_II (Fig. 4B). Moreover, when ANI_I was cultured in ANI_II conditioned medium, the relative amino acid production levels of glutamine, phenylalanine, glycine, threonine, isoleucine, tyrosine, proline, and arginine were less than 1 (*t*-test, *p* < 0.05) in ANI_I (Fig. 4A and Supplementary Fig. 10). This result confirmed a metabolic cooperation related to phenylalanine, glycine, and threonine, where the ANI_II group increased the production of these amino acids and the ANI_I group utilised these amino acids (Supplementary Table 4).

**Fig. 5:**
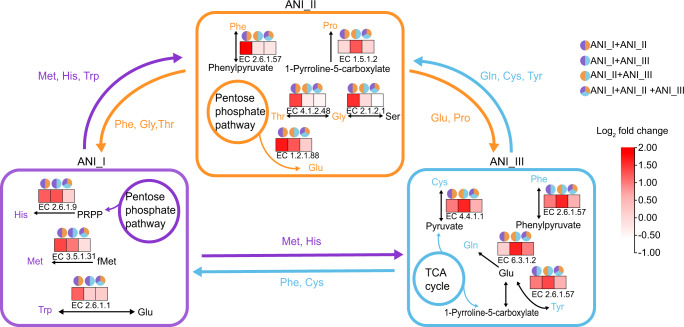
Construction of metabolic cooperation scheme related to amino acid between three genotypic groups of *L. acetotolerans*. The transcription of the last gene in the biosynthesis pathway of related amino acid is shown in the frame of different genotypic groups, and the heatmap is shown using the log_2_ fold change value of transcriptional data in coculture compared to monoculture. The small pie chart above the heatmap indicates the coculture mode.

We then confirmed other metabolic cooperation between different genotypic groups. The ANI_I group increased the production of methionine and histidine for the ANI_III group, and the ANI_III group acted as a producer of phenylalanine and cysteine for the ANI_I group. The ANI_II group acted as a producer of glutamate and proline for the ANI_III group, and ANI_III acted as a producer of glutamine, cysteine and tyrosine for the ANI_II group (Figs. 4 and 5, Supplementary Fig. 10A–C, Supplementary Table 4). In general, we revealed metabolic cooperation related to amino acids between different conspecific genotypic groups within *L. acetotolerans* species, which would improve fitness of *L. acetotolerans* species.

## Discussion

With methodological and conceptual advances, an increasing number of methods have become available to classify microbial strains [38, 39]. ANI classification has appeared to offer finer resolution between conspecific strains [29]. Microorganisms within the same species usually show ≥95% ANI among themselves [40]. However, a good cut-off for strain-level differentiation is still difficult to determine. It was suggested that strains with higher than 99% ANI would constitute an ecotype based on the comparison of 70 closely related bacterial genomes [41]. It was suggested that bacteria with 2% ANI divergence can be ecologically homogeneous based on metagenomic analysis of the bacteria in Trout Bog Lake, Wisconsin [42]. To establish an optimal ANI level for intraspecific interaction studies, we classified 9 isolates into different ANI groups according to different cut-off values, and found that 99.5% might be a good cut-off level for strain classification when studying microbial intraspecific interactions. It indicated that *L. acetotolerans* might interact across groups defined with 99.5% ANI. This level can be referenced for strain classification within species in other microbial ecosystems. Most bacterial species contain strains with different genomic characteristics [43–45], and strain diversity is considered to be functional important for microbial communities [46, 47]. *L. acetotolerans* is a representative species in food fermentations; it has a small genome size (1.49–1.62 Mb) but a large coding density (0.83–0.88) among all reported Lactobacillaceae (Supplementary Fig. 11), indicating high genetic diversity in this species. Therefore, it would be more efficient to reveal intraspecific interactions among diversified genotypic groups within this species. In addition, this work showed that different phylogenetic groups within this species can regulate each other’s biosynthesis of amino acids, and consumed the responsive amino acids. It might be a novel kind of ecological facilitation [48].

Researchers have reported that relatedness plays important roles in microbial interactions and hence influences the assembly of the microbiota [49]. Many studies have shown that closely related species/strains tend to compete for the same resources [3, 50, 51]. However, others suggest that the increase in microbial relatedness drives the evolution of cooperation [52]. This work indicated that most closely related microbes might cooperate. The fitness of *L. acetotolerans* increased with the increase in phylogenetic similarity of the coculture partner within Lactobacillaceae (ANI: 65.8% to 69.2%). *W. paramesenteroides* was the exception in this family, which might be related with its highest average ANI divergence with *L. acetotolerans*. This result indicated that the phylogenetic distance could influence the microbial interactions. As a result, it might be useful to divide the phylogenetic distance into different ranges to analyse the effect of relatedness on microbial interactions.

It was reported the genotypic richness affected the community performance [53]. For example, increasing intraspecific genotypic richness of the resident *Pseudomonas fluorescens* community decreased the invasion success of *Serratia liquefaciens* [54], and genotypic richness was positively related with the primary production [55]. The genome-level difference among different genotypic groups might be related to the microbial evolution within the species [56–58], and would lead to dependence of different genotypic groups within the species. However, we cannot analyse the effect of genotypic group number on the population fitness, because we only obtained three genotypic groups. We would analyse the effect of intraspecific diversity on species traits in the natural microbial community in the future.

This work revealed that genotype group classification would be efficient for analysing intraspecific interactions and showed a novel kind of ecological facilitation related to amino acids occurred between different genotypic groups of *L. acetotolerans*. This result indicated that intraspecific interactions are equally as important as interspecific interactions for the assembly and stability of microbial ecosystems. These results could be beneficial for regulating the microbiota in food fermentation and other natural microbial ecosystems.

## Supplementary Information

Supplementary Material

Supplementary Table 1

Supplementary Table 2

Supplementary Table 3

Supplementary Table 4

## Data Availability

The raw sequence data from the whole–genome sequencing and transcriptomic sequencing of *L. acetotolerans* strains were submitted to the DNA Data Bank of Japan (DDBJ) under the accession numbers PRJDB15432 and PRJDB15434, respectively. The raw sequence data from the whole–genome sequencing of 10 bacterial strains were submitted to DDBJ under the accession number PRJDB15433. The accession number of genome sequence of *B. amyloliquefaciens* CGMCC 12593 was CP011252.
