# Supplementary material for: Metabolic cooperation between conspecific genotypic groups contributes to bacterial fitness
Source: ISME Commun. 2023 Apr 28;3:41. doi: 10.1038/s43705-023-00250-8 (PMC10147913; doi:10.1038/s43705-023-00250-8)
Supplement: Supplementary file 1 — Supplementary Material [file 43705_2023_250_MOESM1_ESM.docx]

**Supplementary** **figures**


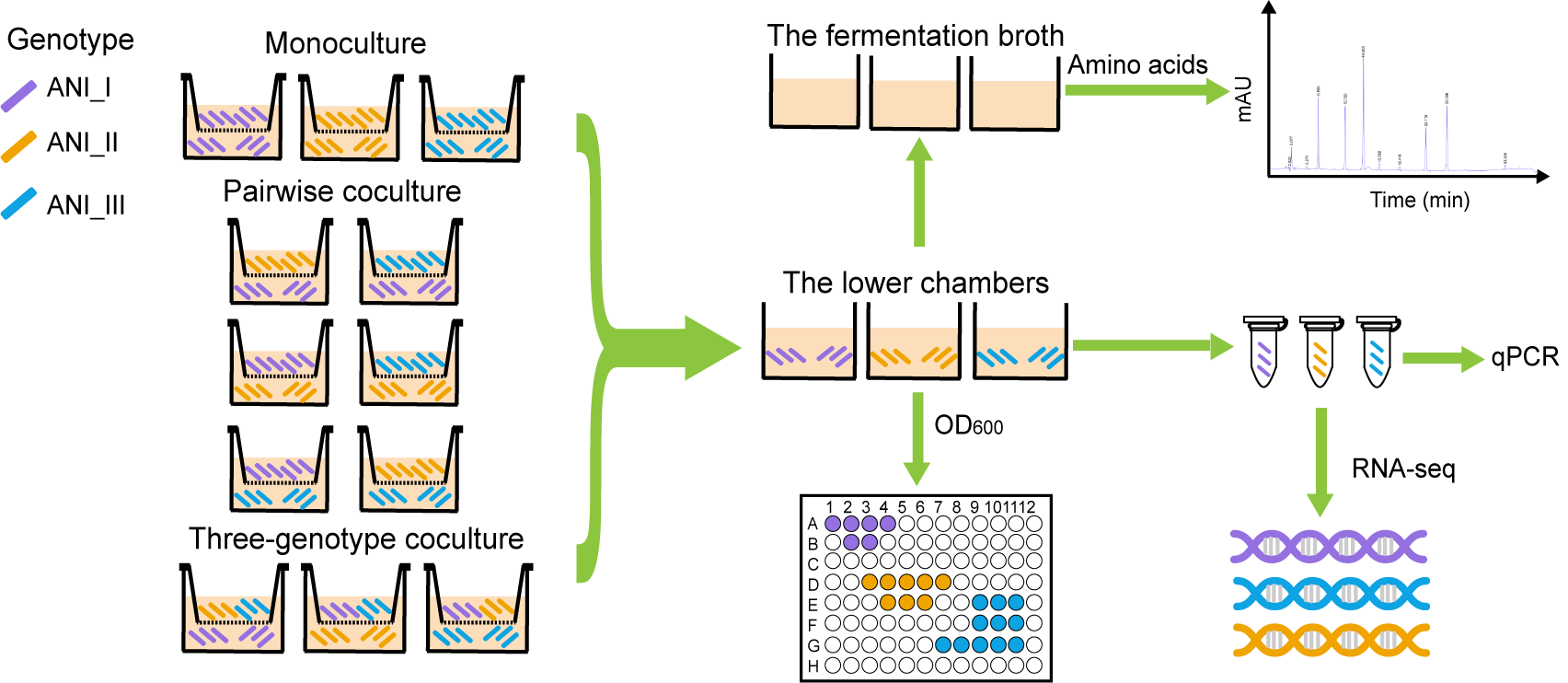


**Supplementary Fig. 1 The design of mono- and coculture in Transwell plate.** Monoculture and coculture experiments were performed in 2 mL of chemically defined medium in Transwell plate under static and anaerobic conditions at 30 °C for 8 days. The bacterial solution was collected for analysis of biomass, amino acid, and transcription. In monoculture, one genotypic group was inoculated in both upper and lower chambers. In pairwise coculture, the two genotypic groups were separately inoculated in the upper and lower chambers. In three-genotype coculture, two genotypic groups were inoculated into the upper chamber, and the other genotypic group in the lower chamber. To keep the culture condition consistent for different genotype groups, we only collected bacterial solution in the lower chamber for analysis of biomass, amino acid, and transcription.


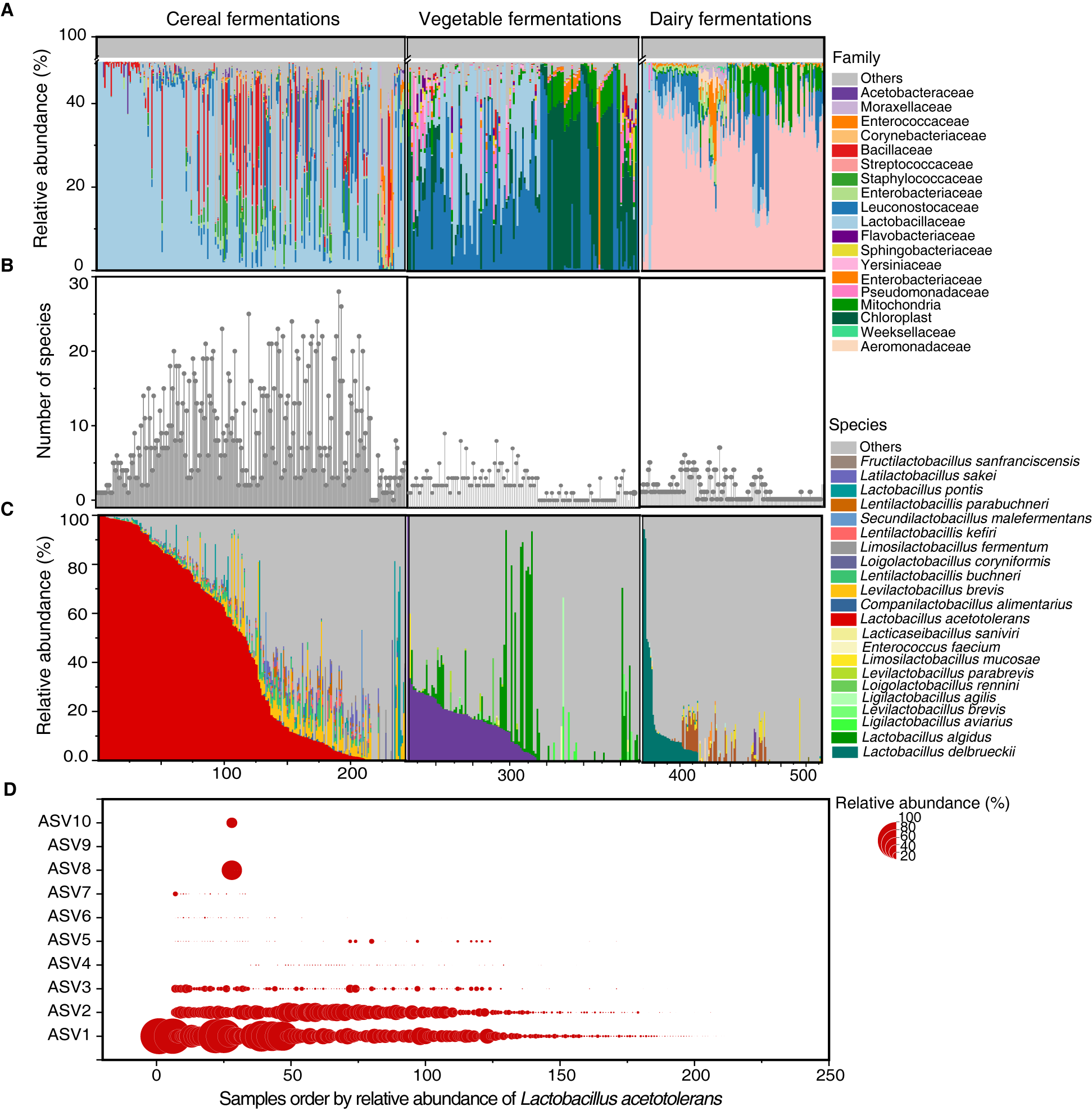


**Supplementary Fig. 2 The distributions of lactobacilli in 558 food fermentation ecosystems.** **A**, Histogram of the relative abundance of bacteria (top 10 families in each sample). Species not included in the top 10 in relative abundance are shown as Others. **B**, The number of Lactobacillaceae species per sample. **C**, The relative abundance of species (top 10 in each sample) in Lactobacillaceae. Species not included in the top 10 in relative abundance are shown as Others. **D**, Bubble plots of the relative abundance at the ASV level of *L. acetotolerans* in cereal fermentations based on the V3-V4 region of bacterial 16S rRNA (mean = 490 bp). The samples were ordered by relative abundance of *L. acetotolerans* in cereal fermentations, vegetable fermentations and dairy fermentations, respectively.


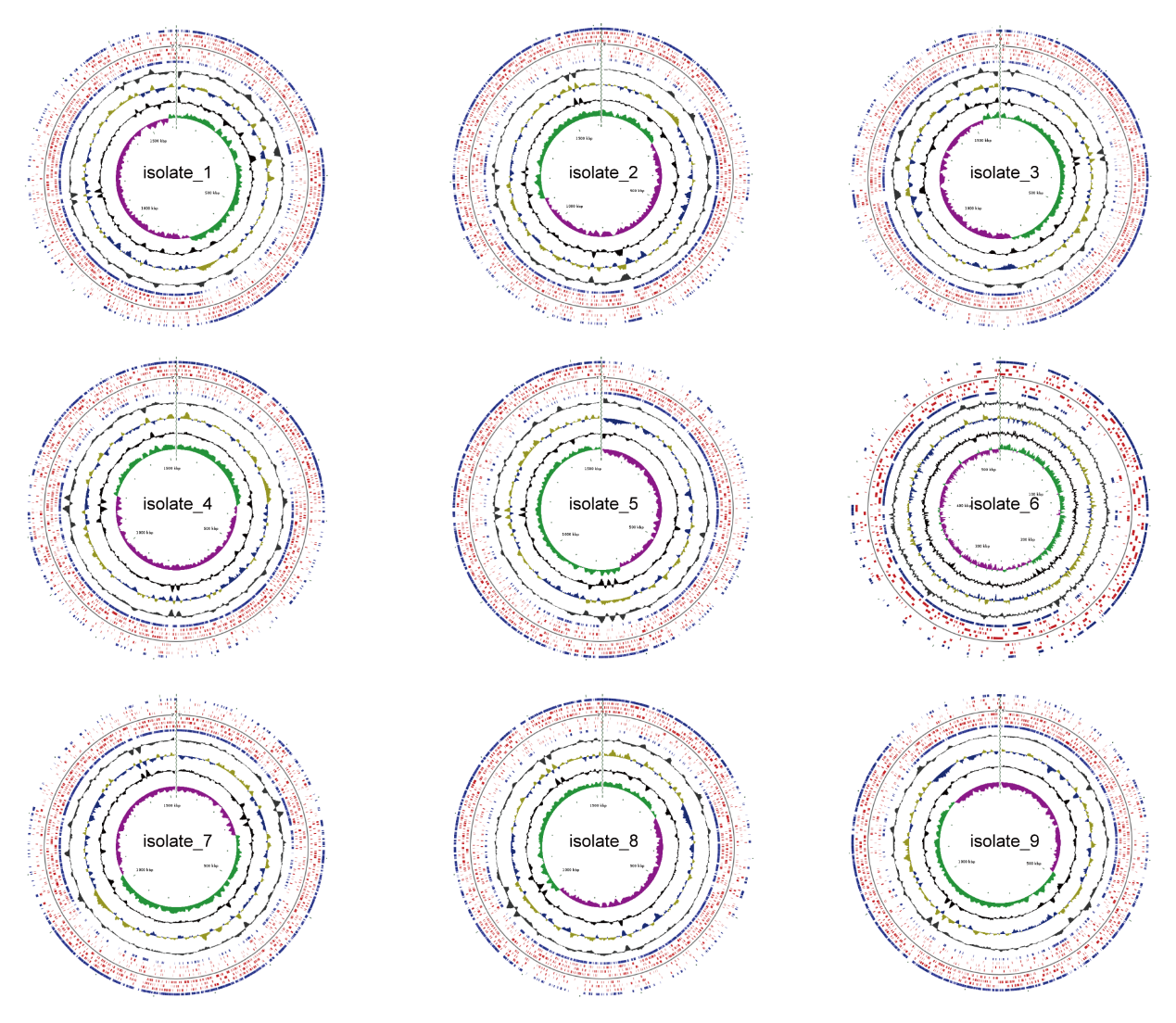


**Supplementary Fig. 3 The genomes of 9 *L. acetotolerans* isolates.** The outer circle shows the coding sequence regions. The second and third circles mark tRNA and rRNA, respectively. The fourth circle shows open reading frames. The fifth to tenth circles show the AT-content, positive AT-skew, negative AT-skew, GC-content, positive GC-skew and negative GC-skew, respectively.


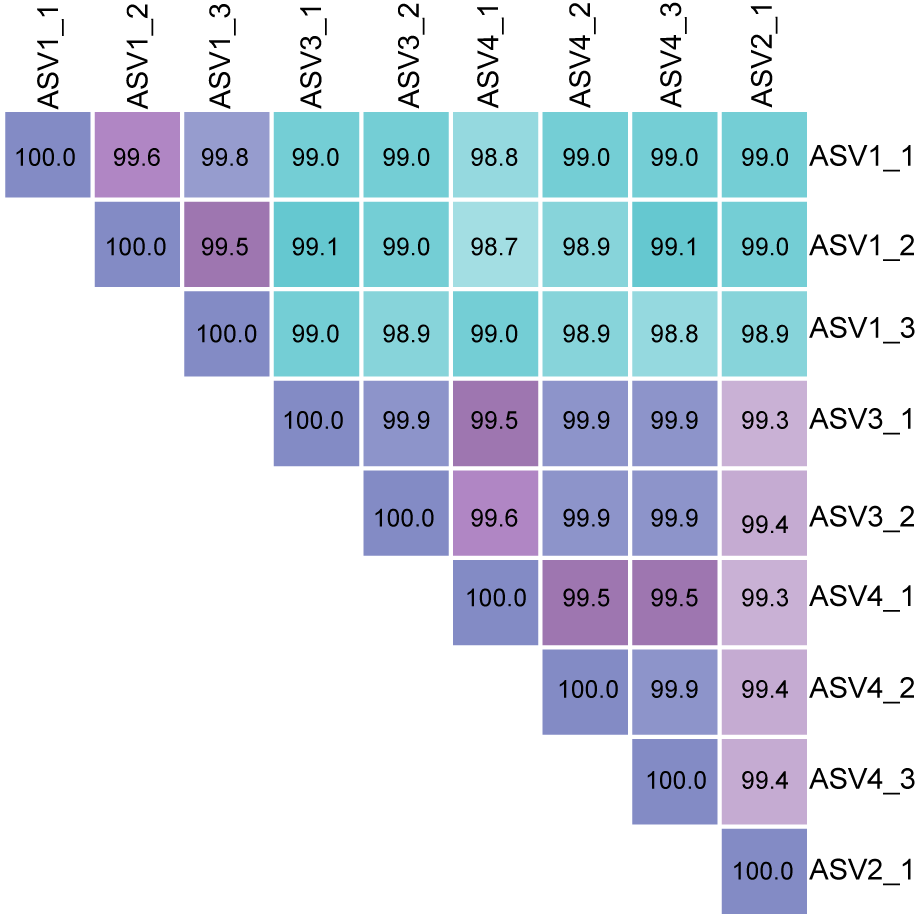


**Supplementary Fig. 4 ANI matrix of 9 sequenced genomes of *L. acetotolerans* isolates.**


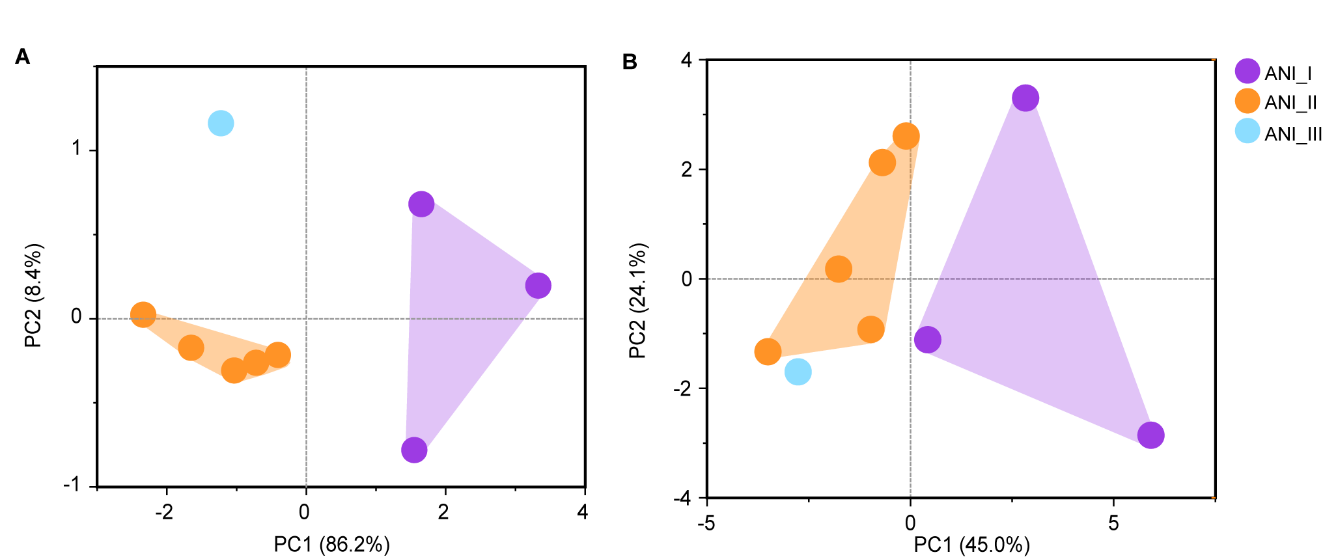


**Supplementary Fig.** **5 Differences of** **gene contents of COG in three ANI groups compared by principal component analysis.** The variation in the gene contents of all CAZyme category (**A**) and amino acid transport and metabolism (**B**).


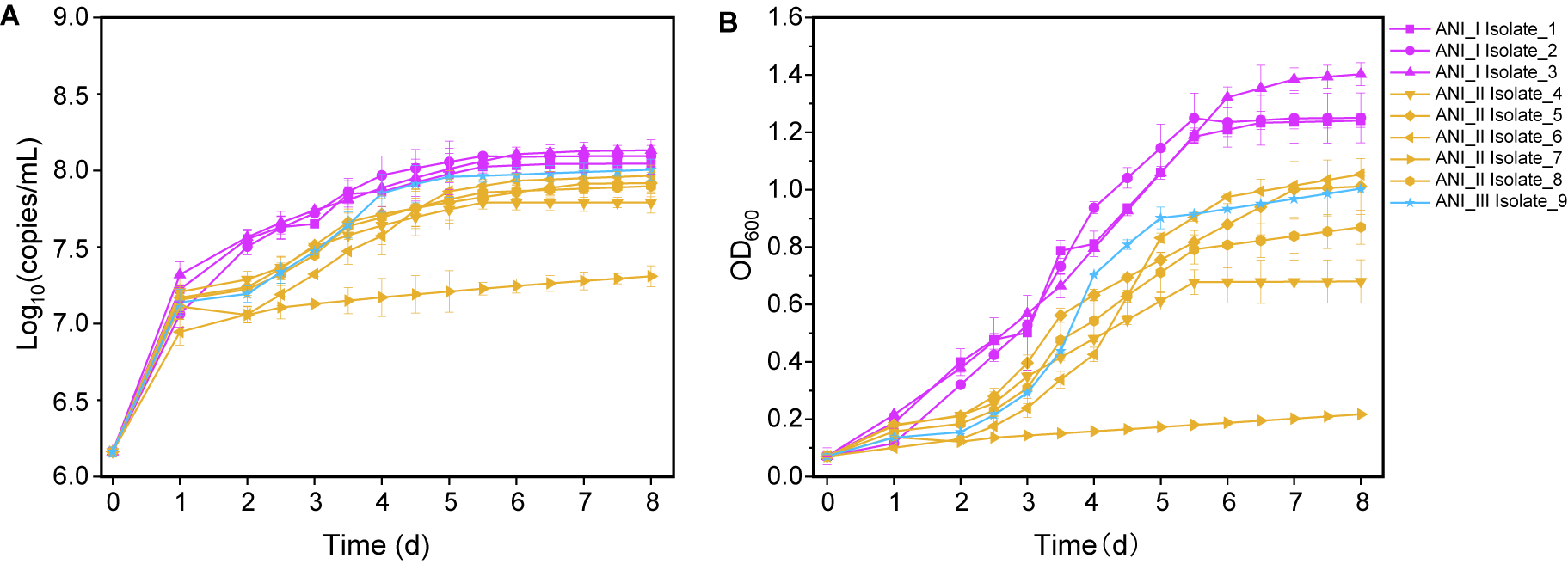


**Supplementary Fig. 6 The growth curves of three ANI groups.** Biomass was measured by qPCR (**A**) and OD_600_ (**B**). Cultures were performed in 1.5 mL of chemically defined medium in 96-well plates under static and anaerobic conditions at 30 °C for 8 days. Error bars indicate the standard deviation from four biological replicates.


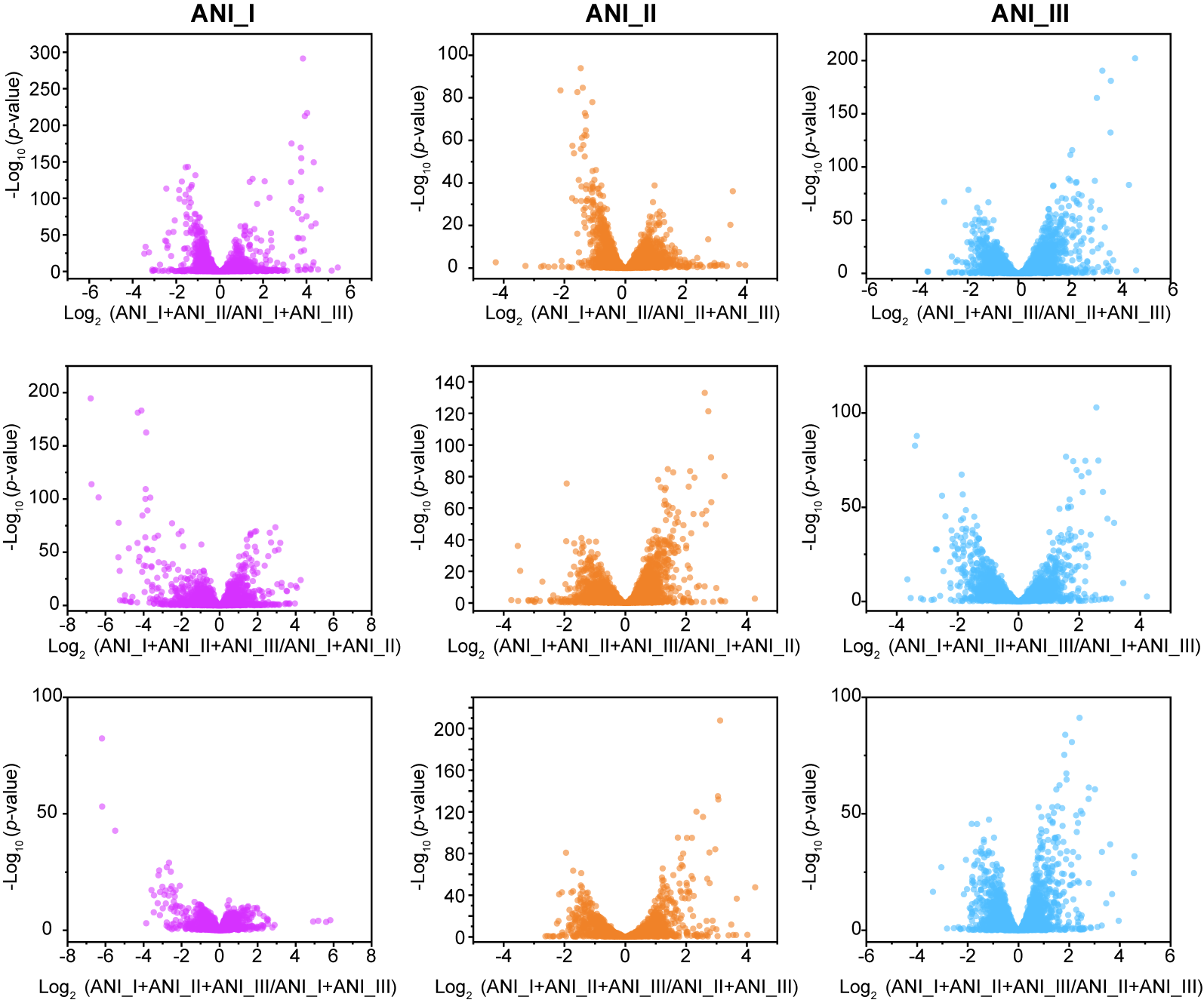


**Supplementary Fig. 7 Volcano plots of transcriptional data in pairwise cocultures and three-genotype cocultures compared to different cocultures.** Each dot indicates the gene transcription of one isolate in different cocultures. Transcription data were from two coculture experiment groups, one containing isolate_1 (ANI_I), isolate_4 (ANI_II), and isolate_9 (ANI_III) and the other containing isolate_3 (ANI_I), isolate_8 (ANI_II), and isolate_9 (ANI_III). Cultures were performed in 2 mL of chemically defined medium in Transwell plates under static and anaerobic conditions at 30 °C. The bacterial solution was collected on Day 4 for transcription analysis. All data are based on four biological replicates.


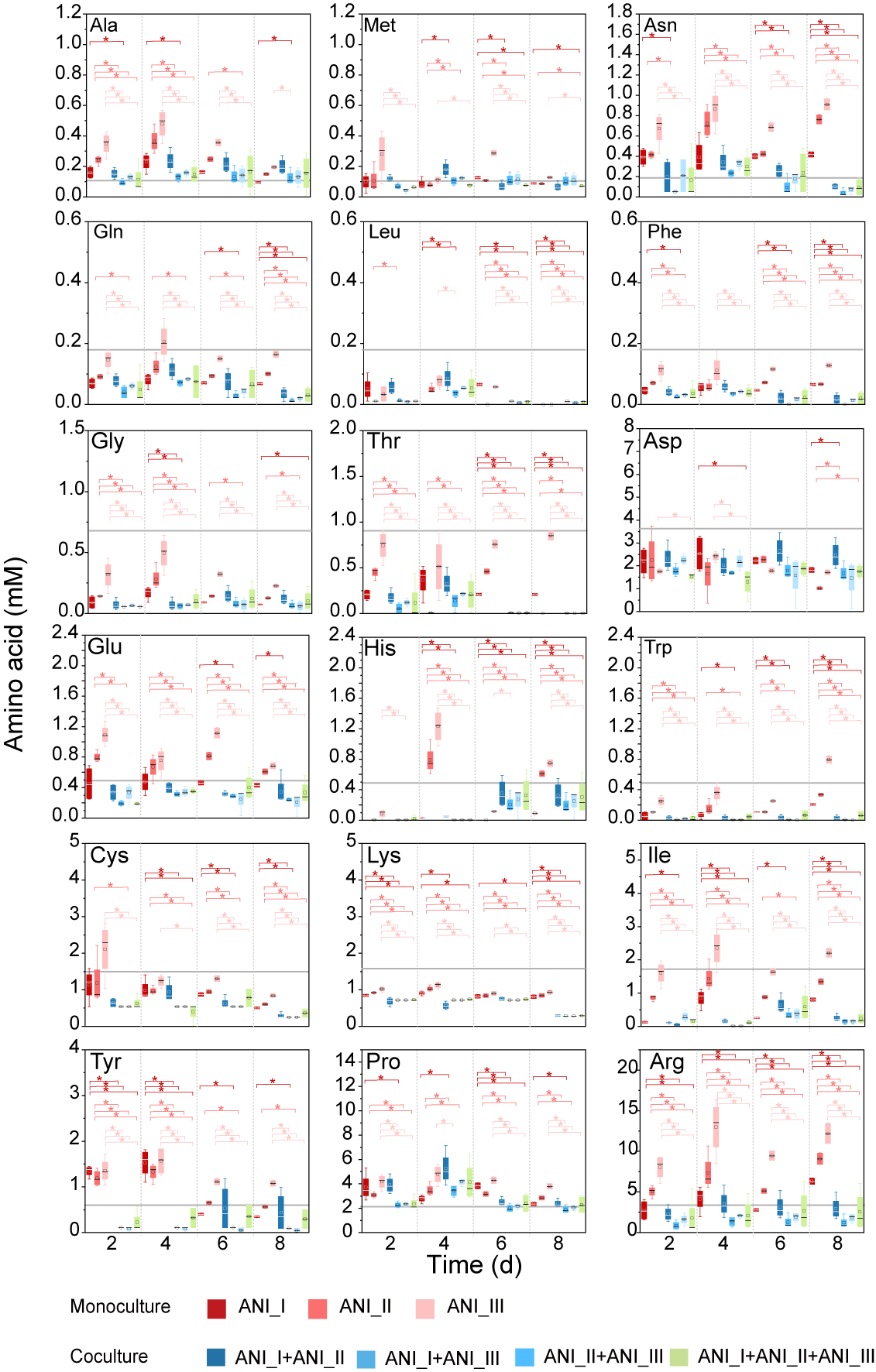


**Supplementary Fig. 8 Concentrations of amino acids in mono- and cocultures of different ANI groups.** The box plots show the concentrations of amino acids. The gray lines in the panels indicate the initial concentration of amino acid in the culture. Data were from two coculture experiment groups, one containing isolate_1 (ANI_I), isolate_4 (ANI_II), and isolate_9 (ANI_III) and the other containing isolate_3 (ANI_I), isolate_8 (ANI_II), and isolate_9 (ANI_III). Cultures were performed in 2 mL of chemically defined medium in Transwell plates under static and anaerobic conditions at 30 °C. The bacterial solution was collected on Days 0, 2, 4, 6 and 8 for amino acid determination. Error bars indicate the standard deviation from four biological replicates. Asterisks indicate significant difference of amino acid concentrations between monoculture and coculture at different time point (*t*-test, *p* < 0.05).


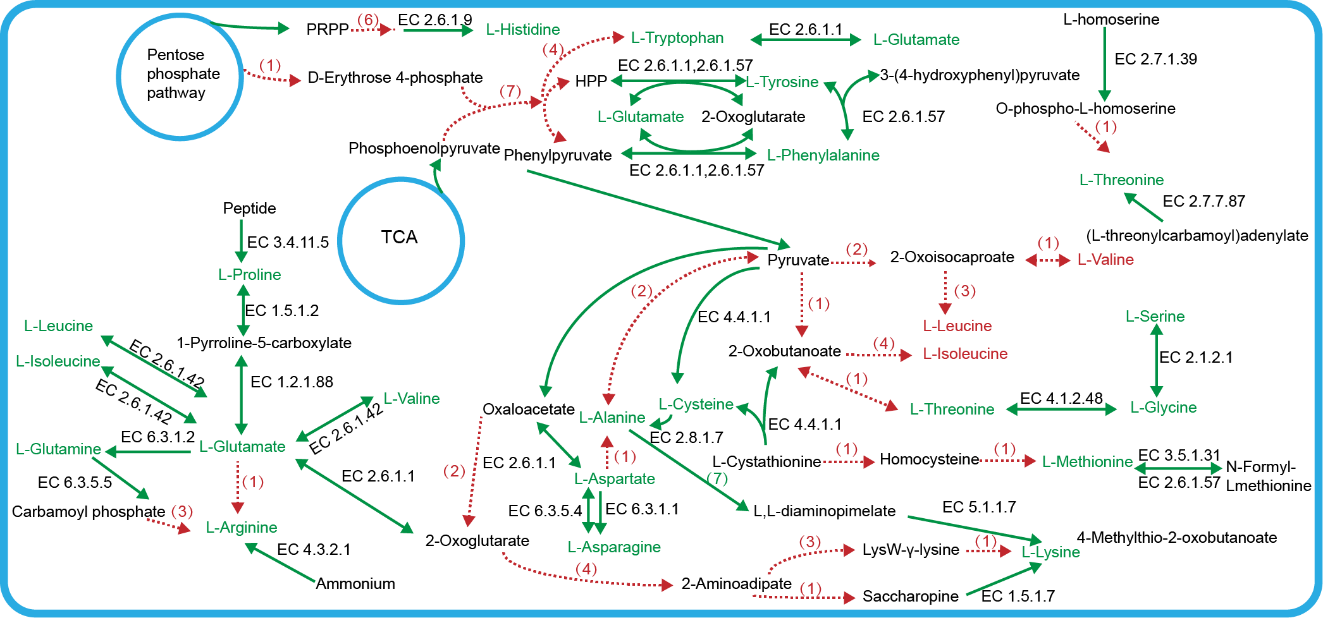


**Supplementary Fig. 9 Construction of amino acid biosynthesis pathway in *L. acetotolerans*.** Metabolic predictions are mainly generated by referring to the KEGG pathway database and MetaCyc Metabolic Pathway Database. Enzymes related with amino acid biosynthesis are used to show the potential activity of *L. acetotolerans.* According to the whole genomes, all 9 isolates have the potential to synthesise 20 amino acids. Solid green arrows indicate the existence of corresponding genes in the pathways, dotted red arrows indicate the miss of corresponding genes in the pathways, and the number in brackets indicate the number of missed genes in the pathway. Amino acids shown in green are those can be synthesised, and shown in red are those that cannot be synthesised in certain pathway by prediction.


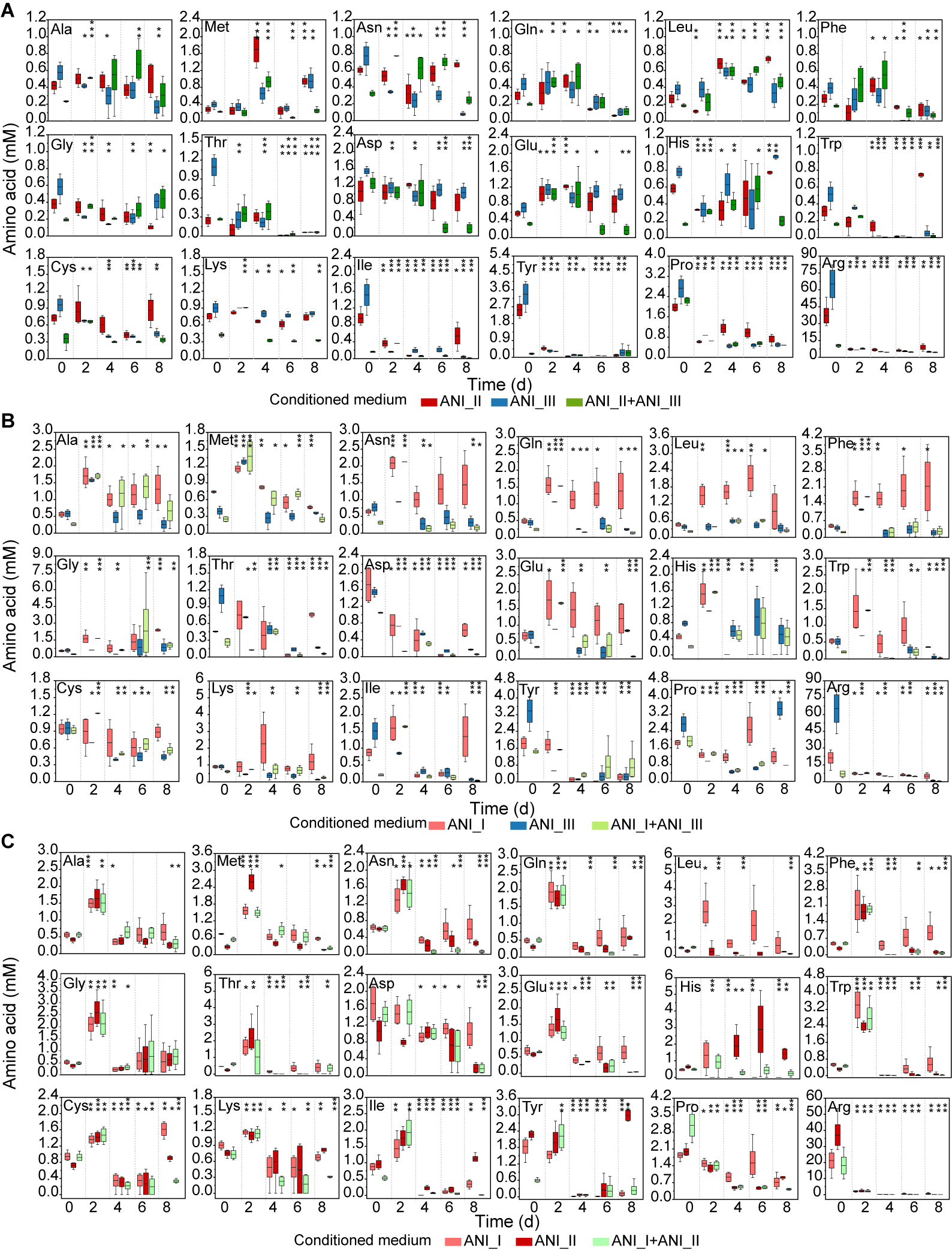


**Supplementary Fig. 10 Concentrations of amino acids of different ANI groups in culture experiments with conditioned medium.** The amino acid concentrations of ANI_I (**A**), ANI_II (**B**) and ANI_III (**C**). The box plots show the concentrations of amino acids. The data were from two coculture experiment groups, one containing isolate_1 (ANI_I), isolate_4 (ANI_II), and isolate_9 (ANI_III) and the other containing isolate_3 (ANI_I), isolate_8 (ANI_II), and isolate_9 (ANI_III). Cultures were performed in 2 mL medium in Transwell plate under static and anaerobic conditions at 30 °C. The bacterial solution was collected on Days 0, 2, 4, 6 and 8 for amino acid determination. Error bars indicate the standard deviation from four biological replicates. Asterisks indicate significant difference of amino acid concentrations on Days 2, 4, 6 and 8, against that on Day 0 (*t*-test, * *p* < 0.05, ** *p* < 0.01, *** *p* < 0.001).


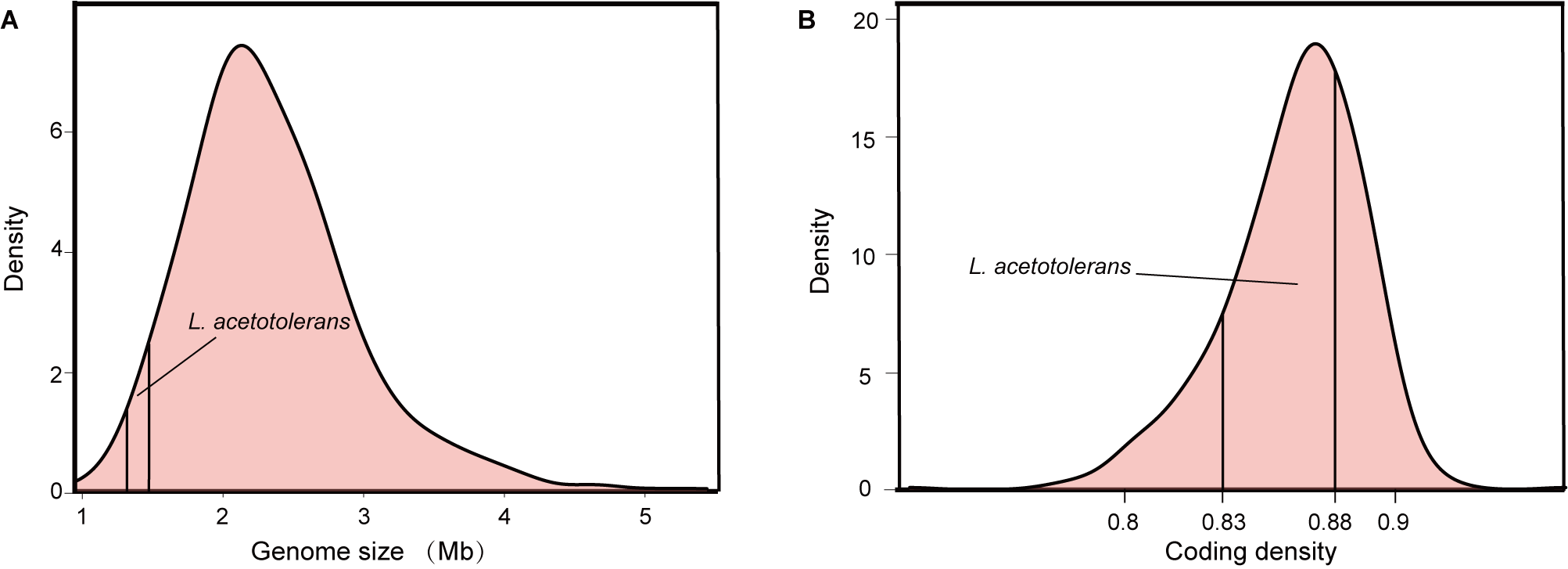


**Supplementary Fig. 11 Genome size range (A) and gene coding density distribution (B) of *L. acetotolerans* among reported Lactobacillaceae.**
